# Prediction of survival and analysis of prognostic factors for hepatocellular carcinoma: a 20-year of imaging diagnosis in Upper Northern Thailand

**DOI:** 10.1186/s12885-023-11429-6

**Published:** 2023-11-04

**Authors:** Nawapon Nakharutai, Imjai Chitapanarux, Patrinee Traisathit, Pimwarat Srikummoon, Suwalee Pojchamarnwiputh, Nakarin Inmutto, Wittanee Na Chiangmai

**Affiliations:** 1 Department of Statistics, Faculty of Science, Chiang Mai University, Chiang Mai, Thailand; 2 Chiang Mai Cancer Registry, Maharaj Nakorn Chiang Mai Hospital, Faculty of Medicine, Chiang Mai University, Chiang Mai, Thailand; 3 Department of Radiology, Faculty of Medicine, Chiang Mai University, Chiang Mai, Thailand

**Keywords:** Hepatocellular carcinoma, Survival rate, Risk factors

## Abstract

**Background:**

To evaluate survival rates of hepatocellular carcinoma (HCC), the Chiang Mai Cancer Registry provided characteristics data of 6276 HCC patients diagnosed between 1998-2020 based on evolution of imaging diagnosis. Evolution can be separated into four cohorts, namely, cohort 1 (1990-2005) when we had ultrasound (US) and single-phase computed tomography (CT), cohort 2 (2006-2009) when one multi-phase CT and one magnetic resonance imaging (MRI) were added, cohort 3 (2010-2015) when MRI with LI-RADS was added, and finally, cohort 4 (2016-2020) when two upgraded MRIs with LI-RADS were added.

**Methods:**

Cox proportional hazard models were used to determine the relation between death and risk factors including methods of imagining diagnosis, gender, age of diagnosis, tumor stages, history of smoking and alcohol-use, while Kaplan-Meier curves were used to calculate survival rates.

**Results:**

The median age of diagnosis was 57.0 years (IQR: 50.0-65.0) and the median survival time was 5.8 months (IQR: 1.9-26.8) during the follow-up period. In the univariable analysis, all factors were all associated with a higher risk of death in HCC patients except age of diagnosis. In a multivariable analysis, elderly age at diagnosis, regional and metastatic stages and advanced methods of imagining diagnosis during cohorts 2 and 3 were independently associated with the risk of death in HCC patients. The survival rate of patients diagnosed during cohort 4 was significantly higher than the other cohorts.

**Conclusion:**

As a significantly increasing survival rate of HCC patients in cohort 4, advanced methods of diagnostic imaging can be a part of the recommendation to diagnose HCC.

## Introduction

According to the World Health Organization (WHO), more than 10.0 million people died from cancer in 2020 [1], of which hepatocellular carcinoma (HCC) is one of the top five causes [2, 3]. In 2020, 8.3 hundred thousand people worldwide died of HCC [1] while Thailand’s National Cancer Institute reported 2,890 new cases of HCC in the same year [4]. HCC is the second-most common cancer in males after colorectal cancer whereas it is the fifth-most common in females after breast, colorectal, cervical, and lung cancers [4].

HCC is commonly caused by chronic hepatitis [5] and viral infection [6]. Moreover, other factors such as chronic alcohol consumption [7–10] increase the risk of contracting HCC by 1.5-3.6 times [11], while smokers are 2.6 times more likely to develop HCC than non-smokers. Hepatitis B infection [8–11], non-alcoholic fatty liver disease [11–13], and cirrhosis [8, 11], as well as exposure to nitrosamines [11], aflatoxin [11, 14, 15], vinyl chloride monomers [11, 16], among other carcinogens [8], increase the risk of contracting HCC. However, early diagnosis and prognosis of HCC with effective and potentially curative treatment for starting cancer treatment as soon as possible can increase the patient’s survival rate [17, 18]. In 2016, 15.5 million patients in the USA survived cancer due to early detection of the disease, and that number was expected to rise to 20.3 million by 2026 [18].

Radiological imaging is one of the most important tools for the early diagnosis of HCC [19]. In recent decades, advancements in radiological imaging technologies, equipment, and contrast agents have revolutionized the way radiologists can detect and diagnose HCC early with greater accuracy [20, 21]. As these technologies have evolved, scientific organizations have also developed an integrated imaging system called the Liver Imaging Reporting and Data System (LI-RADS) to help with the surveillance, diagnosis, staging, treatment, and monitoring of therapeutic response in HCC patients [22]. The first version of LI-RADS (v1.0) was released in 2011 [22]. Since the release of version 1.0, LI-RADS has been continuously updated to include science, technology, and user feedback. Major LI-RADS updates followed in 2013, 2014, 2017 and 2018 [23].

Maharaj Nakorn Chiang Mai Hospital, Faculty of Medicine, Chiang Mai University is a leading tertiary care hospital and cancer treatment and research center in the upper north of Thailand [24]. Every day, a large number of people arrive for diagnosis and treatment [25]. The hospital provides services through a multidisciplinary care team including doctors, nurses, medical technicians, physical therapists, nutritionists, social workers, and pharmacists. Furthermore, there is also a medical school with many professional specialists, medical professors, specialty nurses, and modern medical technology [24]. Therefore, in cooperation with Maharaj Nakorn Chiang Mai Hospital, we can obtain a huge retrospective cohort of HCC patients diagnosed from 1998 to 2018 in order to study the survival rate and examine the factors related to HCC surveillance, including assessing the diagnosis of HCC by using radiological imaging, over the past 20 years.

During this period, methods of imaging diagnosis included ultrasound (US), computed tomography (CT) and magnetic resonance imaging (MRI). To examine the relationship between mortality rate and risk factors associated with these imaging methods, we divided the 20-year period into four periods corresponding to the evolution of these imaging methods in each period. The aim is to utilize the results as a guideline for the use of radiological imaging to diagnose HCC in the future.

## Materials and methods

### Patients and data collection

Clinical individual information for each HCC patient at diagnosis, namely demographics (gender, age, smoking history, and alcohol-use history), cancer characteristics, methods of diagnosis and cohorts, can be obtained from Chiang Mai Cancer Registry, Maharaj Nakorn Chiang Mai Hospital. For cancer characteristics, the data are recorded as the Surveillance, Epidemiology, and End Results (SEER) staging: localized, regional, or metastasis. HCC patients were included in the study if they were diagnosed between 1 January 1998 and 31 December 2018 and followed-up from the date of registration to the end of 2020. This data can cover almost HCC patients in the Upper Northern of Thailand since Maharaj Nakorn Chiang Mai Hospital has been this region’s primary referral and tertiary center from the past to present.

Note that the Chiang Mai Cancer Registry has recorded HCC patients’ data based on the International Agency for Research on Cancer (IARC) CanReg5 tool which does not record the American Joint Commission on Cancer (AJCC) staging system nor the type of HCC cancer. Hence, these characteristics were not available to be analyzed in this study.

### Methods of imaging diagnosis

**Fig. 1:**
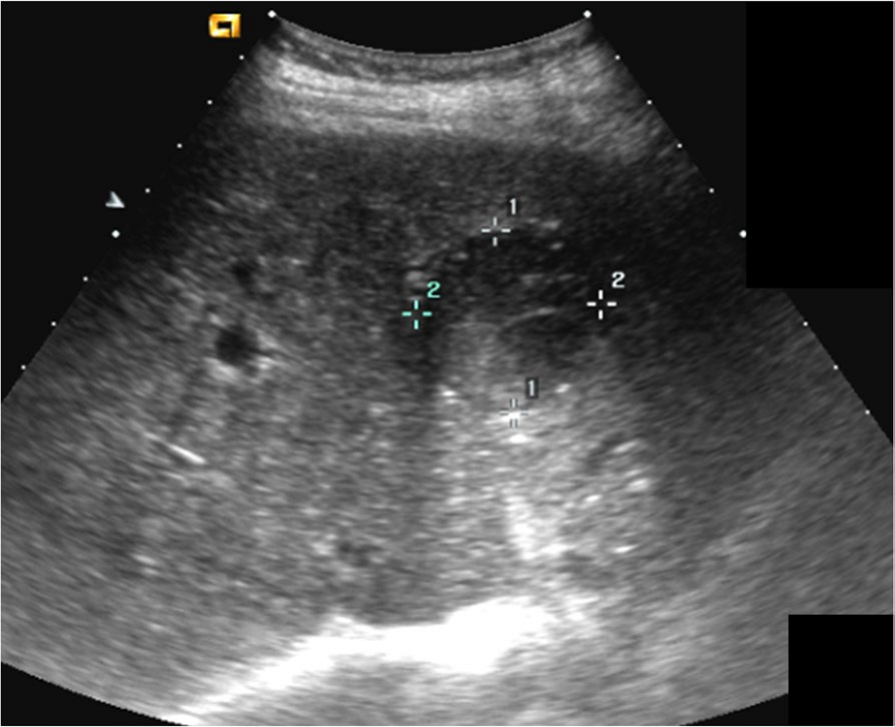
US of the liver shows a well-defined, heterogeneous hypoechoic nodule in left hepatic lobe

US is the most widely accepted method to screen for HCC in high-risk patients [26–28] but it should be performed by a trained professional [29]. HCC is often hypoechoic in nature (Fig. 1). According to the American Association for the Study of Liver Diseases (AASLD) and the European Association for the Study of the Liver (EASL), an ultrasound test every 6 months is recommended for patients with cirrhotic livers [30], while blood tests [27, 31] usually combined with an ultrasound test increase diagnostic accuracy [11].

**Fig. 2:**
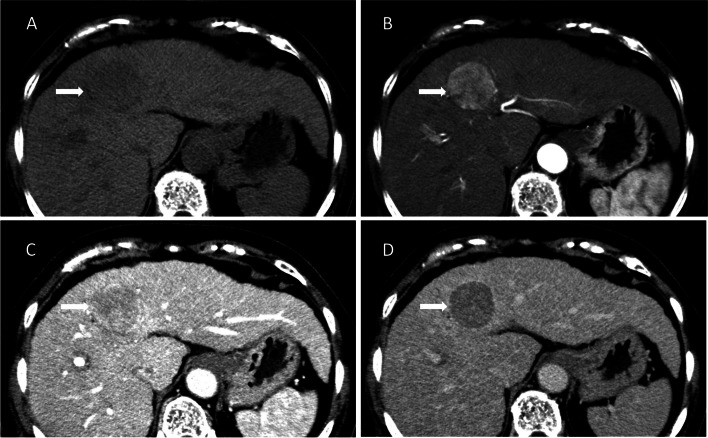
**A** Axial plain CT image shows a round hypoattenuating nodule in left hepatic lobe (arrow). **B**-**D** Axial contrast-enhanced CT in hepatic arterial phase (**B**) shows hyperenhancement and wash-out appearance in portal venous phase (**C**) and delayed phase (**D**)

Dynamic multiphasic CT and MRI are the first diagnostic methods for HCC [32]. Intravenous extracellular iodinated-contrast agents are used for CT, while extracellular gadolinium-based contrast media or hepatobiliary contrast agents are used for MRI. The use of extracellular contrast agents has been recommended since the inception of LI-RADs (v1.0) [22]. HCC is characterized by hyperenhancement in the hepatic arterial phase and wash-out appearance in poral-venous or delayed phases, as compared to normal hepatic parenchyma (Fig. 2). The diagnosis can be made without further histopathological confirmation. Their specificities were 87%-98 and 84%-97%, respectively [32].

**Fig. 3:**
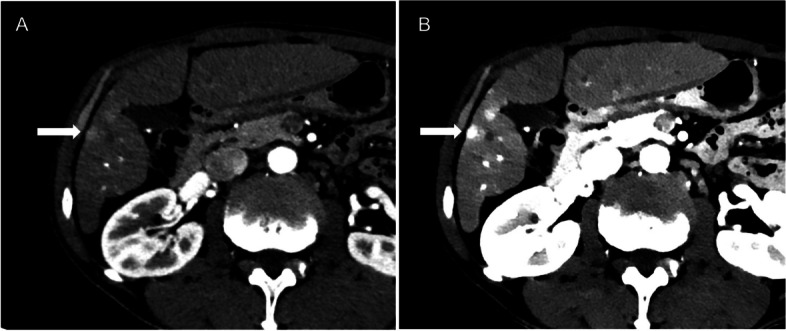
**A** Conventional CT on hepatic arterial phase image depicts faintly small enhancing lesion in segment V of liver (arrow). **B** Monochromatic image viewed at 40 keV from the spectral images derived from dual layer detector CT with greater visibility of lesion (arrow) than conventional routine image

Dual-energy CT (DECT) was an advanced technology that had the potential to improve lesion detection and characterization beyond levels currently achievable with conventional CT techniques [33]. DECT can help to detect small and faint arterial enhancing hepatic nodules by utilizing two different X-ray energies to obtain multiple set of images, allows differentiation of material and tissue composition based on differences in iodine and water densities based on their energy related attenuation characteristics (material density) [33]. The use of advanced DECT has revealed that tissue contrast increases significantly at low energy values, especially for hypervascular lesions [34]. The enhanced nodule compared with surrounding normal tissues is apparent, increasing the visibility of early-stage HCC lesions that are difficult to find in conventional enhanced CT [35] (Fig. 3).

MRI is utilized as the standard problem solving modality for indeterminate lesions [36]. Recently, several functional MRI imaging techniques have been developed to improve the noninvasive assessment of HCC [32]. The most significant techniques are diffusion weighted image (DWI) and hepatobiliary contrast agents [37].

**Fig. 4:**
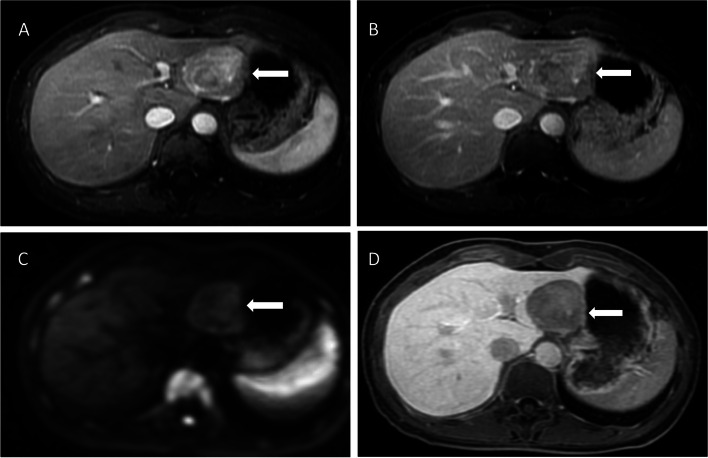
**A** and **B** Contrast-enhanced T1-weighted MR images with hepatobiliary contrast agent show arterial hyperenhancing mass in left hepatic lobe (arrow in **A**) with wash-out appearance on delayed phase (arrow in **B**). **C** Diffusion weighted MR image with high B value shows hyperintense mass compared to the surrounding hepatic parenchyma. **D** Hepatobiliary phase MR image shows hypointense mass compared to the surrounding hepatic parenchyma

DWI is a functional MRI technique that allows quantitative measurements of proton diffusion in tissues [38]. HCC and other types of cancer often have an increased number of cells, thus restricting the diffusion of protons in water [38]. Therefore, most cancers are hyperintense lesions on DWI with a high b value compared to background hepatic parenchyma [39] (as shown in Fig. 4). DWI allows better detection of HCC and better characterizes small lesions [39].

Hepatobiliary contrast agents, including gadobenate dimeglumine (Gd-BOPTA) and gadoxetate disodium (Gd-EOB-DTPA), can provide tumor vasculature and hepatic function information in the same examination [40]. The hepatobiliary contrast agent is absorbed by hepatocytes via OATP8 and decreases progressively during hepatocarcinogenesis [41]. Therefore, most HCCs are hypointense compared to the surrounding hepatic parenchyma during the hepatobiliary phase (Fig. 4).

### Definition of variables

Gender was classified as male or female. Patients’ age at diagnosis was classified into four groups: <50, 50-56, 57-65 and >65. Tumor stage-SEER were classified into three groups: localized, regional, or metastasis. Smoking and alcohol-use history and tissue diagnosis were classified as with or without. Methods of diagnosis was classified into no method or ultrasound, CT, MRI or combined imaging. Table 1 summarises methods of imaging diagnosis with respect to four periods as follows.

**Table 1:** Advanced methods on cohorts

Cohorts	US for	Single-phase	Multiphase	MRI with	MRI with
	screening	CT	CT	extracellular	hepatobiliary
				gadolinium	contrast agents
1	$\checkmark$	CT = 1	-	-	-
2	$\checkmark$	-	CT = 1	MRI = 1	-
3	$\checkmark$	-	CT = 2	MRI = 2	$\checkmark$
4	$\checkmark$	-	CT = 3	-	Upgraded MRI = 2

Cohort 1 (1998-2005): Maharaj Nakorn Chiang Mai Hospital had a single slice CT scanner and could be performed the examination after injecting contrast agent only in the porto-venous phase. This is because a single-slice CT scanner takes time to examine. It is not fast enough to scan during a hepatic-arterial phase in a timely manner.

Cohort 2 (2006-2009): Maharaj Nakorn Chiang Mai Hospital had a multi-slice CT scanner and an MRI machine to perform a faster examination and could be examined both the hepatic-arterial phase and porto-venous phase after the injection of contrast medium.

Cohort 3 (2010-2015): Maharaj Nakorn Chiang Mai Hospital had two multi-slice CT scanners and two MRI machines. Most studies for the characterization of hepatic lesions by MRI were examined with extracellular gadolinium contrast agents according to LI-RADS system (2011) recommendation. Both hepatic-arterial phase and porto-venous phase were performed.

Cohort 4 (2016-2020): Maharaj Nakorn Chiang Mai Hospital had three multi-slice CT scanners (which can perform dual energy techniques) and two upgraded MRI machines. Most studies for the characterization of hepatic lesions by MRI were examined with hepatobiliary contrast agents according to the LI-RADS system (2014) recommendation. The hepatic-arterial phase, porto-venous phase, and hepatobiliary phase were performed.

### Statistical analysis

Continuous data in baseline characteristics were reported as medians and interquartile ranges (IQRs) while categorical data were presented as frequencies and percentages. The follow-up period was calculated from the date of registration at Chiang Mai Cancer Registry to the the date of death for any cause, or to the date of last follow-up censored by using the end of the study period (31 December 2020) for living patients.

The associations between the risk of death among HCC patients and the risk factors (namely, gender, age, tumor stage, smoking history, alcohol-use history, and cohorts) was assessed using Cox proportional hazard models. Kaplan-Meier curves were used to calculate survival rates, and log-rank tests were used to test for significant difference between the survival probabilities of the cohorts. Continuous data were dichotomized using quartiles. The variables with *p*-value <0.20 in the univariate analysis were included in the multivariate analysis via a backward elimination procedure. All analyses were performed using STATA (version 16.0).

## Results

### Baseline characteristics

In total, 6,276 patients (5,020 male and 1,256 female) enrolled between January 1998 and December 2020. The median age of diagnosis was 57.0 years (IQR: 50.0-65.0). During a period of study, there are 5,385 HCC patients died from all causes. The median duration of follow-up was 5.7 months (IQR: 1.9-24.0). During the follow-up period, the median survival time was 5.8 months (IQR: 1.9-26.8).

**Fig. 5:**
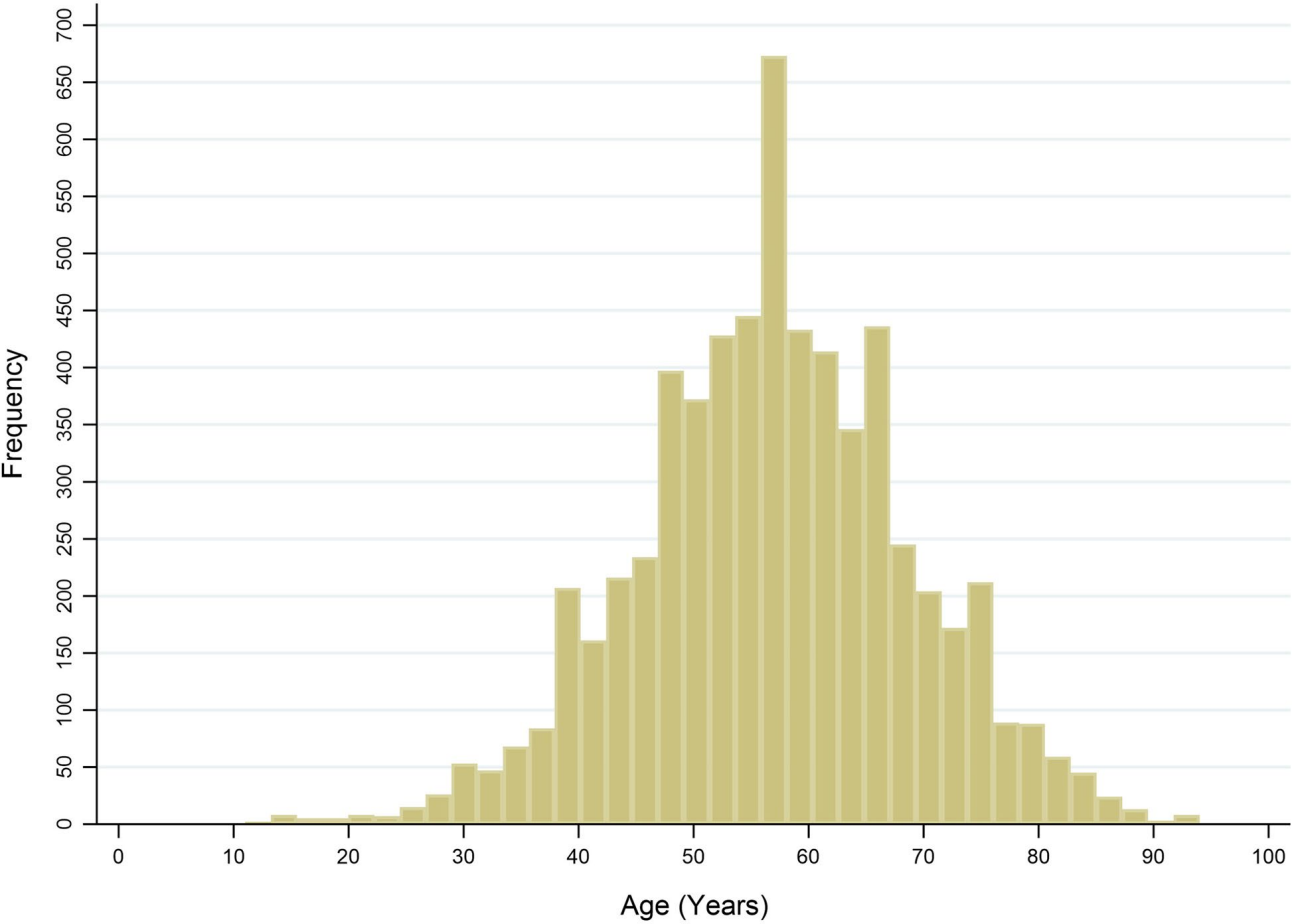
Histogram of age at diagnosis

Table 2 summarises baseline clinical characteristics of these patients. Even though the age at diagnosis trend to be equally distributed among classified groups, the highest frequency of the age at diagnosis was 57.0 - 58.0 years (as shown in Fig. 5). For the HCC tumor staging, the most common stage of HCC patients was 74.3% for localized, while regional and metastasized stages were 7.8% and 17.9%, respectively. Despite a lot of missing history of smoking and alcohol-use, there are 53.3% of patients reported a history of smoking and 67.4% reported a history of alcohol-use. Only 18.7% approached to tissue diagnosis. Methods of diagnosis were separated into four groups: 28.4% for no method or ultrasound, 62.5% for CT, 3.1% for MRI and 6.0% for combined imaging.

**Table 2:** Baseline characteristics of 6276 HCC patients during 1998-2020

Characteristics (missing)	Patients ($\%$)
Gender	
Male	5020 (80.0)
Female	1256 (20.0)
Death	
No	891 (14.2)
Yes	5385 (85.8)
Age of diagnosis (24)	
< 50	1543 (24.7)
50 - 56	1458 (23.3)
57 - 65	1832 (29.3)
> 65	1419 (22.7)
Tumor stages (653)	
Localized	4176 (74.3)
Regional	439 (7.8)
Metastasized	1008 (17.9)
History of smoking (2197)	
No	1903 (46.7)
Yes	2176 (53.3)
History of Alcohol-use (2030)	
No	1318 (32.6)
Yes	2863 (67.4)
Tissue diagnosis	
No	5100 (81.3)
Yes	1176 (18.7)
Methods	
No method or Ultrasound	1784 (28.4)
CT	3921 (62.5)
MRI	194 (3.1)
Combined imaging	377 (6.0)
Cohorts	
Cohort 1 (1998-2005)	1080 (17.2)
Cohort 2 (2006-2009)	995 (15.8)
Cohort 3 (2010-2015)	2365 (37.7)
Cohort 4 (2016-2020)	1836 (29.3)

Table 3 shows the number of patients and overall survival (OS) percentages on different stages of cancer and cohorts. In general, OS percentages of HCC patients in the localized stage was higher than the other stages. We found that OS percentages of HCC patients were gradually increasing throughout cohorts 1 to 4 regardless of cancer stages. This can be inferred that improving technology in methods of imaging diagnosis can bring an increasing of OS percentages of HCC patients for all cancer stages.

**Table 3:** Number of HCC patients and overall survival (OS) percentages on different stages of cancer and cohorts

Cohorts	Patients (%)					
	Localized		Regional		Metastases	
	1-year OS	3-year OS	1-year OS	3-year OS	1-year OS	3-year OS
1	186 (22.8)	105 (12.9)	18 (13.5)	6 (4.5)	8 (9.9)	5 (6.2)
2	251 (39.0)	134 (20.8)	17 (11.0)	3 (1.9)	14 (10.6)	2 (1.5)
3	685 (46.3)	37 (26.1)	47 (11.2)	17 (4.1)	23 (19.5)	12 (10.2)
4	717 (58.1)	491 (39.8)	47 (15.6)	23 (7.6)	37 (34.3)	20 (18.5)

### Risk factors associated with death

Table 4 presents the results of the univariable and multivariable analyses for identifying the risk factors for death in HCC patients. In the univariable analysis, male, regional and metastatic stages, a history of smoking or alcohol-use and cohorts 1, 2 and 3 were all associated with a higher risk of death in HCC patients (all *p*-values <0.001). On the other hand, different ranges for age at diagnosis are not associated with a higher risk of death. We use *p*-values $\le$0.20 to identify clinical characteristic in the univariable to be further added to the multivariate model. We found that gender, age at diagnosis, tumor stages, history of smoking and alcohol-use and cohorts were included in the multivariate model.

In the multivariate model, we found that age at diagnosis at >65 years, regional and metastatic stages and cohorts 2 and 3 were independently associated with the risk of death in HCC patients (all *p*-values <0.001). Specifically, the metastatic and regional stages were associated with a higher risk of death with the first and the second highest adjusted hazard ratios (aHR) = 3.09 (95% CI: 2.81-3.40) and 2.20 (95% CI: 1.92-2.52), respectively. Cohort 2 (aHR = 1.54; 95% CI: 1.38-1.73) and cohort 3 (aHR = 1.32; 95% CI: 1.22-1.44) were also associated with a higher risk of death. However, being male, history of smoking or alcohol-use were not independently associated with the risk of death in HCC patients.

**Table 4:** Risk factors associated with death among the HCC cancer patients

Characteristic	Patients		Univariable analysis		Multivariable analysis	
	Survived	Death	HR (95% CI)	*p*-value	aHR (95% CI)	*p*-value
	($n=891$)	($n=5385$)				
Gender						
Female	202	1054	Ref		Ref	
Male	689	4331	1.13 (1.06-1.21)	<0.001	1.00 (0.90-1.11)	0.957
Age at diagnosis						
< 50	207	1336	Ref		Ref	
50 - 56	207	1251	0.91 (0.84-0.98)	0.012	0.94 (0.84-1.05)	0.260
57 - 65	284	1548	0.91 (0.85-0.99)	0.014	1.12 (1.01-1.24)	0.037
> 65	183	1236	0.99 (0.91-1.07)	0.741	1.28 (1.15-1.44)	<0.001
Tumor stages						
Localized	742	3434	Ref		Ref	
Regional	24	415	2.00 (1.80-2.21)	<0.001	2.20 (1.92-2.52)	<0.001
Metastasized	37	971	2.36 (2.19-2.54)	<0.001	3.09 (2.81-3.40)	<0.001
History of smoking						
No	426	1,477	Ref		Ref	
Yes	306	1,870	1.28 (1.20-1.38)	<0.001	1.18 (1.07-1.30)	0.001
History of alcohol-use						
No	293	1090	Ref		Ref	
Yes	454	2409	1.24 (1.15-1.33)	<0.001	1.11 (1.00-1.24)	0.044
Cohorts						
Cohort 4 (2016-2020)	517	1,319	Ref		Ref	
Cohort 1 (1998-2005)	57	1,023	1.93 (1.78-2.10)	<0.001	1.29 (1.06-1.59)	0.013
Cohort 2 (2006-2009)	62	933	1.6 (1.47-1.74)	<0.001	1.54 (1.38-1.73)	<0.001
Cohort 3 (2010-2015)	255	2,110	1.32 (1.24-1.42)	<0.001	1.32 (1.22-1.44)	<0.001

$^*$ CI, confidence interval; *p*-value from partial likelihood ratio test. HR, hazard ratio; aHR, adjusted hazard ratio

### Survival probabilities

**Fig. 6:**
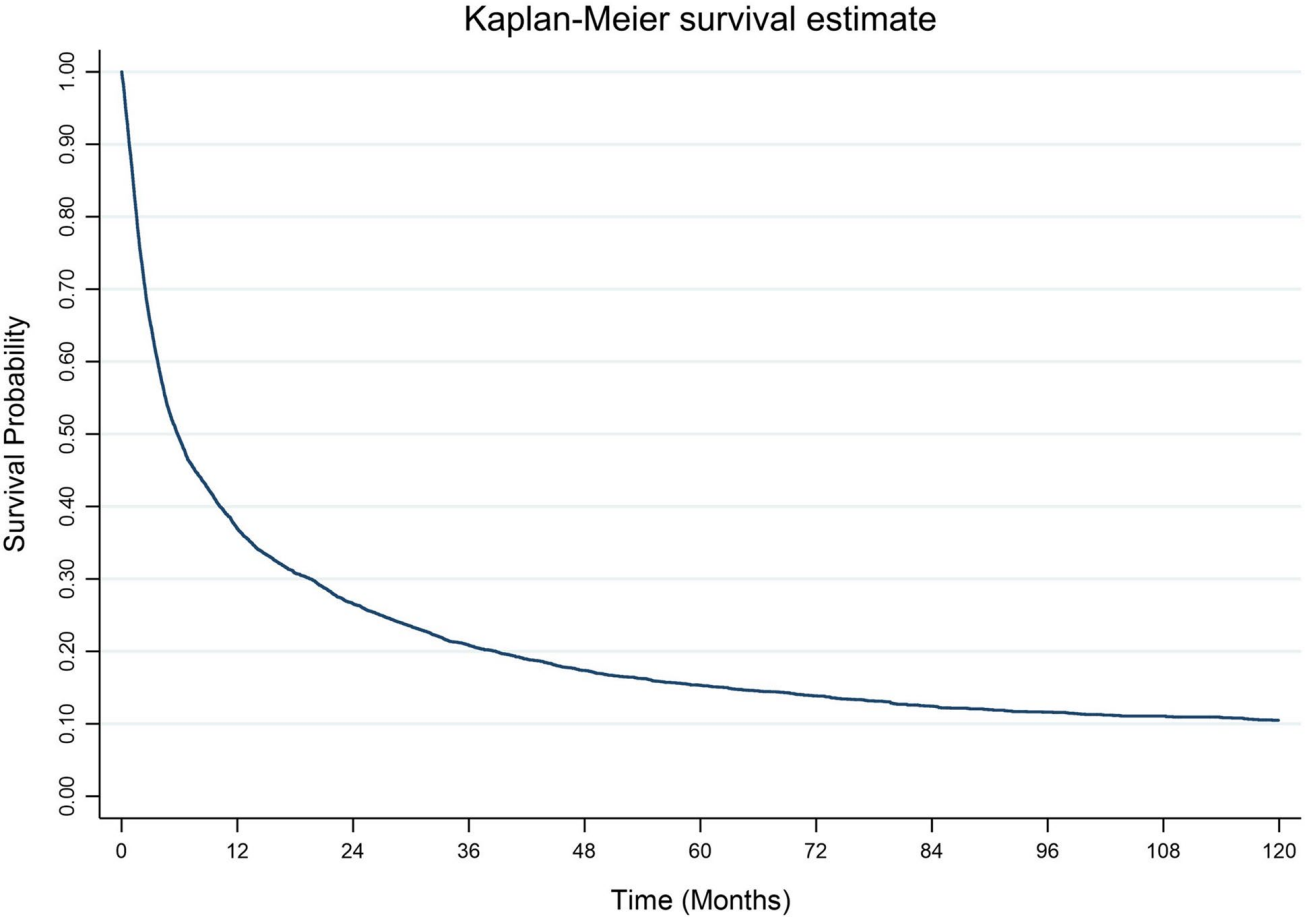
Kaplan-Meier curve of overall survival for HCC patients

The impact of diagnosis time on survival for all cohorts is illustrated in Fig. 6. According to the result, the survival probability approximately dropped to 25% after two years of diagnosis. Throughout the follow-up period after two years since diagnosis, the survival probability gradually decreased to 10%.

**Fig. 7:**
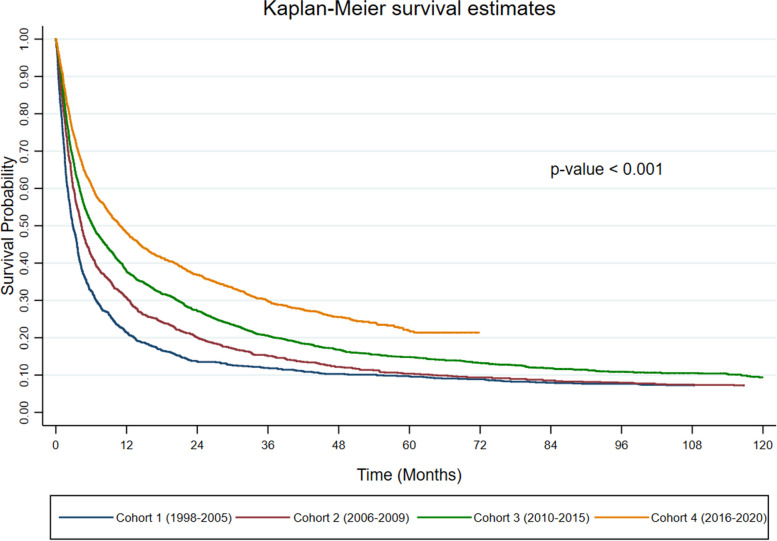
Kaplan-Meier curves of overall survival for HCC patients at different cohorts

The impact of different cohorts on survival time is presented in Fig. 7. It can be inferred that the survival probabilities of HCC patients who are in cohort 4 (2016 - 2020) was significantly higher than the survival probabilities of HCC patients of other cohorts (*p*-value <0.001) Specifically, after the first year of diagnosis, the survival probabilities of HCC patients in cohorts 1, 2, 3 and 4 were approximately 20%, 30%, 40% and 50%, respectively. The survival probability for cohort 4 were 30% after three years of diagnosis while the survival probability for cohorts 1, 2 and 3 dropped to 20% - 10%.

## Discussion

We examined the factors and studied the mortality rate in HCC patients in the Upper Northern of Thailand. We found that in the univariable analyses, being male, smoking history, alcohol-use history are well known mortality risk factors for HCC which are consistent with those from other studies [42–45], but disagree with [46]. Meanwhile regional and metastasized stages are also mortality risk factors for HCC which agree with those from other studies [47, 48]. Compare to advanced method of imaging diagnosis in cohort 4, lacking and unimproved method of imaging diagnosis during cohorts 1, 2 and 3 are also mortality risk factors for HCC in the univariable analyses. For the multivariable analyses, age at diagnosis >65 is associated with a higher risk of death in HCC patients which is consistent with this study [49]. Additionally, regional and metastasized stages and diagnosis during cohorts 2 and 3 are associated with a higher risk of death in HCC patients.

Associated risk of imaging diagnosis can be explained by the development of detection machine in our hospital during the periods. Specifically, during the 1998-2005 period, there were only US and single-slice CT scanners in our hospital but CT scanners were not well developed. The scan could take a long time, and hepatic arterial phase (HAP) could not be performed. Consequently, radiologists were unable to diagnose small or early HCC. However, a formal surveillance guideline was published in 2001, and the surveillance tools are AFP concentration and US [50]. The first non-invasive criteria for HCC diagnosis based on imaging were developed, and the concept of arterial phase hyperenhancement was introduced in the EASL 2001 guideline [50].

By 2005, imaging technology had evolved to include contrast-enhanced ultrasound, MDCT and dynamic MRI [51]. Emerging evidence demonstrated that the dynamic vascular pattern of HCC could be assessed using these imaging modalities, in particular arterial phase hyperenhancement (APHE) followed by washout appearance [51]. Recognizing these advances in technology and new evidence, the AASLD introduced the concept of APHE and washout appearance as a hallmark feature of HCC in the AASLD 2005 guideline [51].

During 2006-2009, our hospital had one multi-slice CT scanner that could perform multiphase CT studies, including hepatic arterial and porto-venous phases. The diagnosis of HCC with hallmark features can be made without biopsy. From 2010-2015, our hospital had two multi-slice CT scanners and two MRI machines. In this period, the LI-RADS system was developed in 2011 for standardized terminology and report of the entire spectrum of liver findings in patients at risk for HCC [52]. After that, the LI-RADS system underwent regular updates in 2013 and 2014 that considered new evidence and technological advances [52]. The LI-RADS 2014 version incorporates imaging criteria for hepatobiliary contrast agents [52].

During 2016-2020, three multi-slice CT scanners with dual-energy techniques and two upgraded MRI scanners were implemented in our hospital with an updated LI-RADS system. A use of hepatobiliary contrast agent permits evaluation of hepatocyte functions, very sensitive for early and small lesions, can help differentiate early HCCs from cirrhosis- associated benign nodules [53]. The use of advanced DECT has revealed an increase in the visibility of early HCC lesions that are difficult to find in conventional enhanced CT [53].

One-year overall survival rates of our patients increased steadily from approximately 20% (1998-2005) to 30% (2006-2009), 40% (2010-2015), and 50% (2016-2020), respectively, over the continuously increasing periods of diagnosis. A similar trend of increased one-year survival rate can be found in the previous studies. For example, one-year overall survival rates increased from 31.5% (1998-2002) to 40.2% (2003-2007), 47.4% (2008-2012), and 51.2% (2013-2015), respectively, over the continuously increasing periods of diagnosis [54]. Another study showed that One-year overall survival rates increased from 14.36% (1997-2001) to 16.51% (2002-2006), 25.32% (2007-2011), and 38.96% (2012-2016) [55]. In addition, the survival improvements over time were significant in all stage-stratified groups but were more pronounced in patients with localized and regional diseases [54]. In patients with localized HCC, one-year overall survival increased from 32.0% in 1988-1992 to 70.4% in 2013-2015 [54]. A significant improvement in one-year survival was also observed for regional HCC (18.2% to 39.1%) [54]. Note that some factors that may be related to the improved survival rate of HCC patients, such as early detection through ultrasound screening and advancements in treatment, were not considered in this study. Therefore, it was not only imaging improvement alone that had an impact on survival rates.

In addition, there are other factors that are related to this improved survival rate of HCC patients, but we cannot include the investigation. For example, the guidelines for HCC management have been updated and revised over the past few decades as new information and evidence emerge. As new imaging modalities, sequences, and contrast agent change and evolve, the evolution of radiological imaging techniques is valuable to detect early HCC [56]. The increased early detection rate of HCC, along with effective and improvement of treatment, can clearly increase the survival rate of patients [56]. In addition, surgery to remove a tumor helps patients survive longer than palliative care or any other treatment [57], and the average post-surgery survival rates are 63%, 29%, 21%, 15%, and 12% after one to five years, respectively [57]. These guidelines for HCC management and treatment can be further investigated in future work.

Nevertheless, our study had usefulness. First of all, we use very large clinical data of six thousands HCC patients diagnosed during 1998-2018 to investigate mortality and survival rates on HCC patients. Even though there are many investigations on risk factors to survival rate of HCC patients [42–45, 47, 48], our work differed from the previous studies as it aimed to show the impact of the evolution of imaging diagnosis technology toward mortality and survival rates on HCC patients. However, other major factors, such as receiving surveillance and treatment factors, have not been considered in this study yet. Therefore, combining with other significant factors may be able to reveal whether implementing advanced technology in imaging diagnosis could increase the survival rate of HCC patients in the future.

## Conclusion

To conclude, the related mortality risk factors for HCC patient were elderly age at diagnosis, regional and metastasized stages and methods of imaging diagnosis before cohort 4, where the hospital was implemented three multi-slice CT scanners and two upgraded MRI machines. In addition, the survival rate of HCC patients in cohort 4 was much higher than the other cohorts. Even though there are many related factors to the survival of HCC patients, these improved methods of imaging diagnosis can successfully increase the survival rate of HCC patients, which can be a part of the recommendation for the further use of advanced imaging methods to diagnose HCC.

## Data Availability

The datasets used and/or analysed during the current study available from the corresponding author on reasonable request.
